# The PLUTO plastidial nucleobase transporter also transports the thiamin precursor hydroxymethylpyrimidine

**DOI:** 10.1042/BSR20180048

**Published:** 2018-03-29

**Authors:** Guillaume A.W. Beaudoin, Timothy S. Johnson, Andrew D. Hanson

**Affiliations:** 1 Horticultural Sciences Department, University of Florida, Gainesville, FL, U.S.A.; 2 Environmental Horticulture Department, University of Florida, Gainesville, FL, U.S.A.

**Keywords:** hydroxymethylpyrimidine, nucleobase cation symporter 1 family, Thiamin

## Abstract

In plants, the hydroxymethylpyrimidine (HMP) and thiazole precursors of thiamin are synthesized and coupled together to form thiamin in plastids. Mutants unable to form HMP can be rescued by exogenous HMP, implying the presence of HMP transporters in the plasma membrane and plastids. Analysis of bacterial genomes revealed a transporter gene that is chromosomally clustered with thiamin biosynthesis and salvage genes. Its closest *Arabidopsis* homolog, the plastidic nucleobase transporter (PLUTO), is co-expressed with several thiamin biosynthetic enzymes. Heterologous expression of PLUTO in *Escherichia coli* or *Saccharomyces cerevisiae* increased sensitivity to a toxic HMP analog, and disrupting PLUTO in an HMP-requiring *Arabidopsis* line reduced root growth at low HMP concentrations. These data implicate PLUTO in plastidial transport and salvage of HMP.

## Introduction

Thiamin diphosphate (ThDP) is an enzyme cofactor required by virtually all forms of life. ThDP-dependent enzymes participate in crucial metabolic reactions that make or break C–C bonds. Plants, fungi, and many prokaryotes synthesize thiamin *de novo* but it is an essential vitamin for animals.

**Figure 1:**
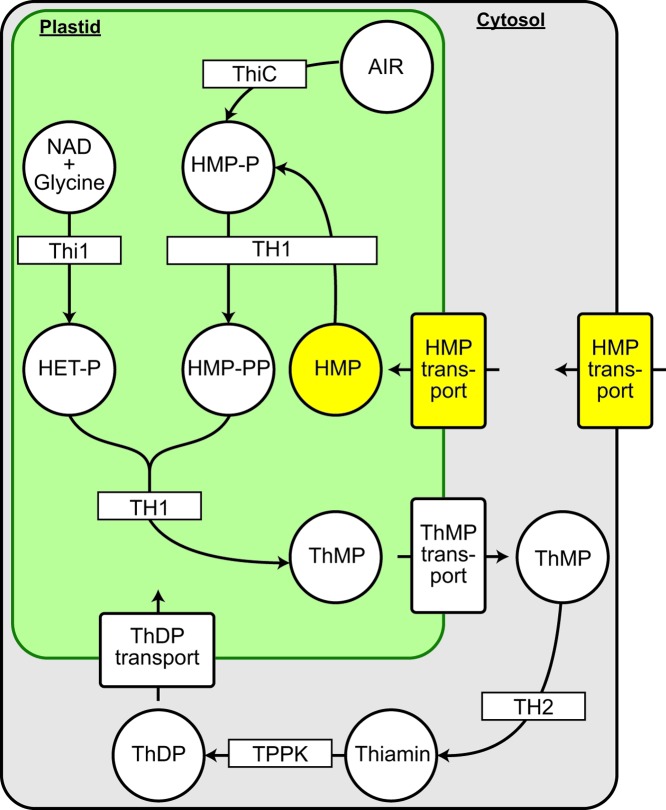
Compartmentation of ThDP biosynthesis in plants and implied transporters The enzymes that use the precursors NAD, glycine, and 5-aminoimidazole ribotide (AIR) to form ThMP are localized exclusively in plastids. The two enzymes that convert ThMP to ThDP are in the cytosol. *Arabidopsis py* (*ThiC*) mutants can be rescued by supplementation with exogenous HMP, implying the presence of HMP transporters in the plasma membrane and the plastid envelope.

Thiamin is composed of hydroxyethylthiazole (HET) and hydroxymethylpyrimidine (HMP) moieties. These are synthesized separately as hydroxyethylthiazole monophosphate (HET-P) and hydroxymethylpyrimidine pyrophosphate (HMP-PP) and then condensed to form thiamin monophosphate (ThMP) (Figure 1). In plants, the enzymes responsible for these reactions are found solely in the plastid [1]. The final conversion of ThMP to ThDP occurs in the cytosol [1–3].

Most of the thiamin biosynthetic enzymes in *Arabidopsis* have been discovered using forward genetics. Mutants deficient in synthesis of the pyrimidine (*py*) or thiazole (*tz*) moieties, or thiamin itself (*th-1, th-2*) were isolated approximately 50 years ago [4], with the last of the corresponding enzymes having been cloned only recently [2]. Exogenous supplementation of a thiamin precursor or thiamin itself rescued the mutant phenotype, allowing identification of the specific defect in each case.

The thiamin and precursor concentrations used in these exogenous supplementation experiments are minute [4], implying the existence of as-yet unidentified plasma membrane transporters for uptake of thiamin or its precursors into the plant. Moreover, to be incorporated into thiamin, HMP must be imported into the plastid from the cytosol, implying the existence of a plastidial HMP transporter (Figure 1). This transporter also remains to be identified.

In the present study, we identified a candidate transporter that is co-expressed with thiamin-related genes in *Arabidopsis* and whose prokaryotic homologs cluster with known thiamin biosynthetic genes. We show that expression of this transporter in *Escherichia coli* or *Saccharomyces cerevisiae* increases sensitivity to a toxic HMP analog, and that knocking out this transporter in an HMP-requiring *Arabidopsis* mutant results in a root growth defect when the level of HMP supplied is lowered.

## Materials and methods

### Bioinformatics

Nucleotide and amino acid sequences were from GenBank or SEED [5]. A taxonomically diverse set of bacterial and archaeal genomes (>1000) were selected and analyzed using SEED tools. Transcriptional network analysis was done using GeneMANIA [6] restricting the data sources to ‘co-expression’ only and using the following genes as queries: *At5g03555, At2g29630, At1g22940, At5g54770, At1g02880, At2g44750, At3g24030, At5g32470, At3g16990, At5g19460, At5g19470, At1g76730, At5g48970, At3g21390*. The names and descriptions of these genes are given in Supplementary Table S1.

### Chemicals

Reagents and chemicals were from New England Biolabs, Fisher Scientific or Sigma–Aldrich except for HMP ((4-amino-2-methyl-5-pyrimidinyl)methanol), oxy-HMP (oHMP; 5-(hydroxymethyl)-2-methylpyrimidin-4(1H)-one), which were purchased from Ark Pharm (Arlington Heights, IL) and [^3^H]thiamin hydrochloride (20 Ci/mmol, 1 mCi/ml in 1:1 ethanol:water) from American Radiolabeled Chemicals (Saint Louis, MO).

### Synthesis of [^3^H]HMP

Briefly, [^3^H]thiamin (30 µCi, 1.5 nmol) was dried *in vacuo* and then hydrolyzed by *Bacillus subtilis* TenA_C in 250 µl as described previously [7]. The reaction was then acidified by adding 25 µl of concentrated H_3_PO_4_ diluted to 500 µl with water and applied to Strata™-XL-AW column (Phenomenex, Belmont, CA) pre-washed with methanol, and pre-equilibrated with 100 mM potassium phosphate, pH 1.1. After sample application, the column was washed with 100 mM potassium phosphate, pH 1.1 and then methanol. HMP, HET, and unreacted thiamin were eluted with methanol:concentrated ammonium hydroxide (9:1) and dried *in vacuo*. The dried eluate was then dissolved in 20 µl of water and applied to a silica G60 F254 thin-layer chromatography (TLC) plate (0.1 mm thickness) (EMD Millipore), and developed with acetonitrile:water (4:1), pH 7.85. The radioactive HMP band (*R*_f_ 0.41) (well separated from thiamin, *R*_f_ 0.085 and HET, *R*_f_ 0.73) was scraped off, extracted thrice with water and lyophilized, yielding 0.56 nmol of [^3^H]HMP (4.45 µCi, 37% yield).

### Expression constructs and strains

The pTAQ-PLUTO expression plasmid with the N-terminal signal peptide removed [8] was used without modification. The empty vector (EV) was obtained by digestion of pTAQ-PLUTO with KpnI and XhoI, blunted with *E. coli* T4 DNA polymerase and ligated with T4 DNA ligase, generating pTAQ-EV. These were then transformed into *E. coli* strain JD23420 lacking the endogenous uracil transporter *uraA*. In order to make a PLUTO *S. cerevisiae* expression plasmid similar to that described previously [9], PLUTO was amplified from pTAQ-PLUTO (forward primer, 5′–3′: ACTG*CTCGAG*AAAAAATGACCGGCTCAGAAATTAATG; reverse primer, 5′–3′: ACTG*TCTAGA*TTACAAAAGCGGATGTGAAGA), digested with XhoI and XbaI and ligated into pRS1024 [10] previously digested with XhoI and SpeI to generate pRS1024-PLUTO. The EV was obtained similarly to pTAQ-MAC-EV except that pRS1024 was digested with XhoI and SpeI, generating pRS1024-EV. The *S. cerevisiae fur4* knockout strain Y03158 (MATα; ura3Δ0; leu2Δ0; his3Δ1; lys2Δ0; YBR021w::kanMX4; Euroscarf, Köhlerweg, Germany) was transformed with the full length *ura3* amplified from *S. cerevisiae* strain S288C (GE Healthcare) (forward primer, 5′–3′: GAGTGAAACACAGGAAGACCAG; reverse primer, 5′–3′: GTTTTGTTCTTGGAAACGCTG) using the LiAc method [11] to generate a uracil prototroph. This strain was then transformed with either pRS1024-PLUTO or pRS1024-EV.

### *E. coli* and *S. cerevisiae* growth experiments

*E. coli* was grown in M9-glucose medium supplemented with 50 µg/ml kanamycin at 37°C, with shaking at 220 rpm. *S. cerevisiae* was grown in synthetic complete medium without uracil and leucine at 30°C, with shaking at 250 rpm. Single colonies (three of each strain) were used to inoculate 2 ml of medium and grown until the OD_600_ reached 2–3. These were then used to inoculate, to an OD_600_ of 0.05, another 2 ml of media with or without oHMP and in the case of *E. coli*, with or without IPTG. These were then grown for 24 h, and their OD_600_ measured.

### *E. coli* transport assays

*E. coli* harboring pTAQ-PLUTO or pTAQ-EV were grown and induced with IPTG as described previously for uracil transport assays [8] and resuspended to an OD_600_ of 10 in M9-glucose medium. Uptake assays were initiated by combining 500 µl of the cell suspension and 500 µl of M9-glucose containing [^3^H]HMP (25 nM, 0.1 nCi). Aliquots (50 µl) were removed immediately and after 2, 5, 10, and 20 min, passed through a pre-rinsed 0.45 µm cellulose nitrate filter (Whatman), and washed twice with 2 ml of M9-glucose medium. [^3^H]HMP uptake of the retained cells was then determined by liquid scintillation counting.

### *Arabidopsis PLUTO py* double mutant lines

The homozygous PLUTO T-DNA *Arabidopsis* line WiscDsLox419Co3 [9] (stock number CS854962) and the *py-1* (*ThiC*) mutant [7] (stock number CS3491) were crossed. F_1_ seeds of the *py-1* mutant line were grown on ½ Murashige and Skoog (MS) medium [12] plates supplemented with 2% (w/v) sucrose, 0.1% (w/v) MES, pH 5.7, and 0.6% (w/v) phytagel. Only plants containing the T-DNA insertion, verified using 3′ (5′–3′: GAATTTCCTTCCCTGTCCTTG) and 5′ (5′–3′: CCGGAGGGACTCAAAGAGTAC) gene-specific primers and a T-DNA-specific primer (p745; 5′–3′: AACGTCCGCAATGTGTTATTAAGTTGTC) were selfed and F_2_ seeds grown on the above medium supplemented with 100 µM thiamin. True-breeding lines were selected by verifying the *py* phenotype [4,7] on thiamin-free medium and the lack of a wild-type copy of PLUTO using the above primers.

### *Arabidopsis* root growth experiments

Seeds were sown on plates of the above medium without thiamin, supplemented with various concentrations of HMP. They were then left in darkness at 4°C for 3 days before being placed vertically under fluorescent lights (Sylvania F40/CWP 40W cool-white plus, 100–150 µmol photons m^−2^ s^−1^) on a 12-h-light/12-h-dark cycle at 22°C. Images were captured 6 days after germination and root length was determined using ImageJ [13].

## Results

### Prediction of PLUTO as an HMP transporter

**Figure 2:**
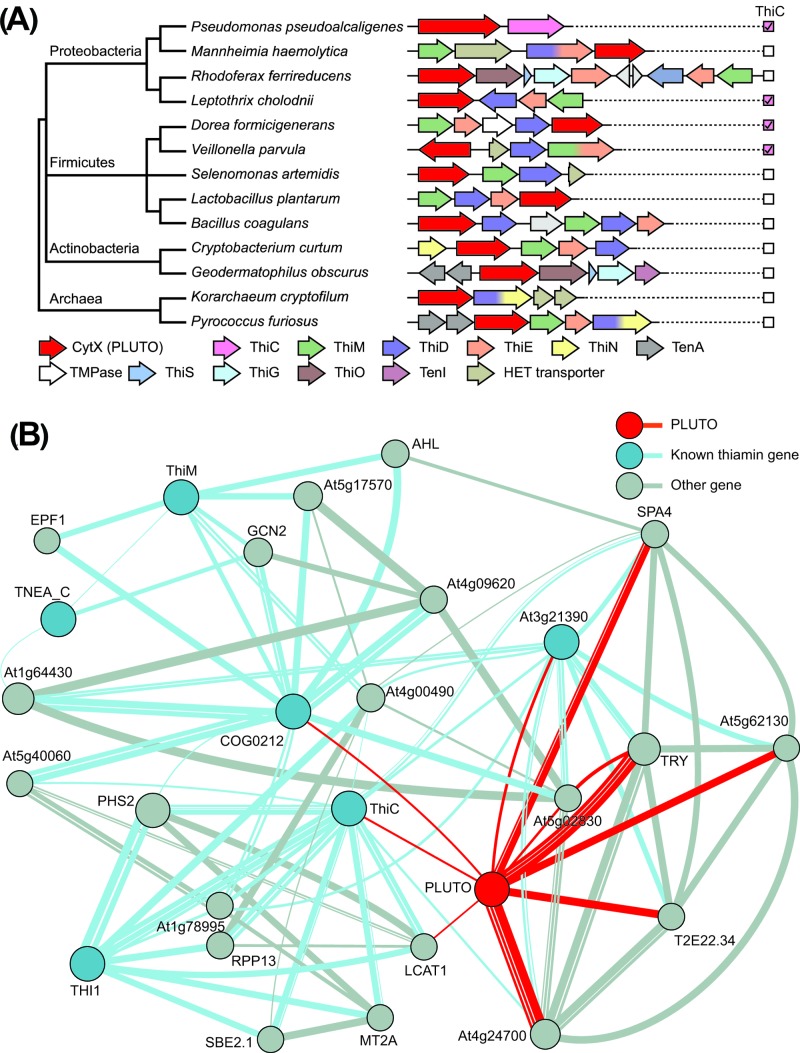
PLUTO and its prokaryote homolog CytX are associated with thiamin synthesis in prokaryotes and *Arabidopsis* (**A**) Clusters of thiamin synthesis (Thi) genes in four different phyla include CytX, a homolog of PLUTO. Note that many of these organisms lack ThiC, whose presence in the genome is denoted by a checked pink box. This implies that PLUTO homologs may transport HMP in prokaryotes. The light gray genes in *Rhodoferax ferrireducens* and *Bacillus coagulens* encode proteins that are unrelated to thiamin metabolism. (**B**) Co-expression network analysis using GeneMANIA reveals an association between PLUTO and several thiamin genes in *Arabidopsis*. Red, blue, and gray lines indicate an association between PLUTO, known thiamin genes and other genes, respectively. The weight of the lines and sizes of the nodes represent the confidence of the association, while the numbers of lines indicate how many sources of expression data underlie the association. Locus tags and descriptions of co-expressed genes are listed in Supplementary Table S1.

Exploiting the fact that genes operating in the same pathway are often clustered on bacterial chromosomes [14], we used the SEED database and its tools [5] to search for uncharacterized transporter genes adjacent to known thiamin biosynthetic genes. This approach identified genes encoding a candidate transporter, CytX, which has also been assigned a putative role in HMP transport by others [15]. The CytX-thiamin association is found in several different configurations and in at least four phyla, making it very robust [16] (Figure 2A). The presence of CytX in organisms that lack the HMP synthesis enzyme ThiC (Figure 2A) suggest that CytX transports HMP rather than HET or thiamin.

We used *Pyrococcus furiosus* CytX as a BLASTp query to identify an *Arabidopsis* homolog. PLUTO (At5g03555), a member of the nucleobase cation symporter 1 (NCS1) family, was the bi-directional best hit with 23% identity. This lies within the ‘twilight zone’ of protein sequence pair alignments (20–35%), within which function may be conserved [17]. Additional support for PLUTO as a potential HMP transporter came from building a co-expression network with GeneMANIA [6] using PLUTO and the known thiamin biosynthetic and related transport and salvage genes. In a network containing six thiamin-related genes (Figure 2B), PLUTO connects directly to three thiamin-related genes (*ThiC, COG0212*, and *At3g21390*) and to a fourth (*Thi1*) through an unrelated gene (*LCAT1*). Other thiamin genes such as *TPPK* (*At1g02880*), *TH1* (*At1g22940*), and *TenA_E* (*At3g16990*) also used as queries were not included in the network. Taken together, this genomic and transcriptomic evidence implicates PLUTO in thiamin metabolism and specifically in HMP transport.

### Expression of PLUTO in *E. coli* or *S. cerevisiae* causes hypersensitivity to oxy-HMP

We chose to use PLUTO heterologously expressed in both *E. coli* and *S. cerevisiae* as a model because it has been reported to be active in these systems in the transport of nucleobases [8,9]. The *S. cerevisiae* strain was modified to be prototrophic for uracil to avoid competitive inhibition of PLUTO with supplied uracil. We used oxy-HMP (oHMP) as a substrate analog of HMP because it is thought to be incorporated into an inactive form of ThDP, resulting in toxicity and slower growth [18].

**Figure 3:**
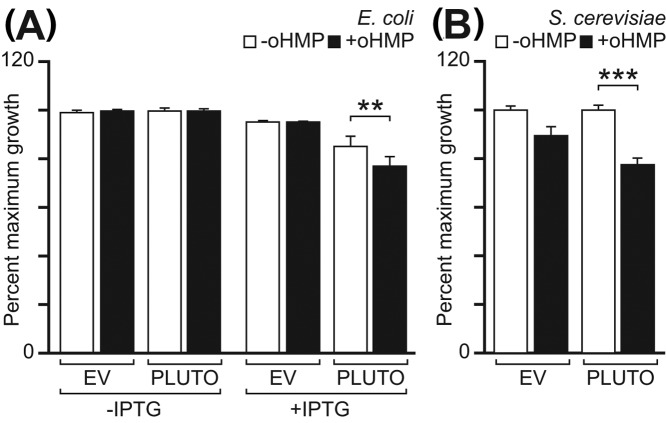
Expression of PLUTO in *E. coli* or *S. cerevisiae* increases sensitivity to the toxic HMP analog oxy-HMP (**A**) *E. coli* cells harboring a PLUTO-expressing plasmid (PLUTO) or the EV were grown in M9-glucose medium at 37°C with or without the addition of 1 mM oxy-HMP (oHMP) or IPTG. A significant decrease in growth upon addition of oHMP was seen only in PLUTO-expressing cells induced by addition of IPTG. (**B**) Likewise, in *S. cerevisiae* with EV, or PLUTO under the control of a constitutive promoter, a significant difference in growth upon addition of 3 mM oHMP was only found in PLUTO-expressing cells. *S. cerevisiae* was grown in synthetic complete medium without uracil and leucine (SC-leu-ura) at 30°C. All cultures were inoculated at an OD_600_ of 0.05 and measured after incubation for 24 h. ‘Maximum growth’ values are relative to the mean OD_600_ of each strain without addition of IPTG or oHMP after 24 h. Data are means of three independent determinations; error bars are the SEM. Significance was determined using a *t*-test, **P<0.01, ***P<0.001.

*E. coli* cells expressing PLUTO showed reduced growth in the presence of 1 mM oHMP, while uninduced cells or those harboring the EV showed no reduction of growth (Figure 3A). Similarly, *S. cerevisiae* expressing PLUTO had significantly less growth after 24 h in the presence of 3 mM oHMP, while no significant change was measured in the empty-vector control (Figure 3B). These data demonstrate that PLUTO can mediate uptake of oHMP.

[^3^H]HMP was synthesized and used in *E. coli* transport assays to attempt to confirm the HMP transport activity of PLUTO. However, due to high endogenous HMP transport activity, no difference between PLUTO-expressing cells and empty-vector controls was detected (Supplementary Fig. S1).

### *Arabidopsis py PLUTO* double mutants have a distinct phenotype rescuable by HMP

**Figure 4:**
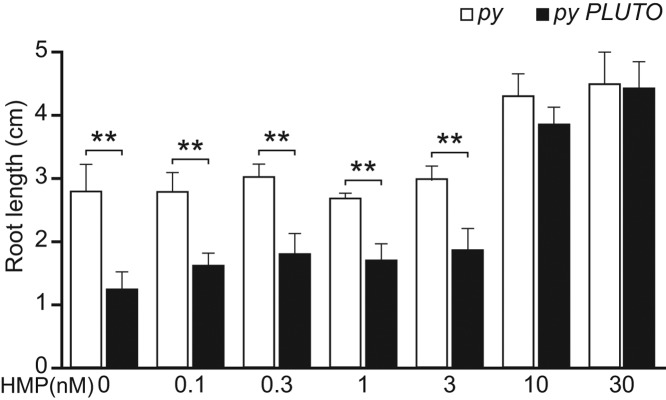
Disruption of PLUTO in the HMP-requiring *py* mutant of *Arabidopsis* leads to a root growth defect that is rescued by supplementing with high concentrations of exogenous HMP *Arabidopsis py* mutants or *py PLUTO* double mutants were grown for 6 days on ½ MS medium with sucrose. The double mutant shows less root growth than the single mutant at HMP concentrations below 10 nM. Representative plants are shown in Supplementary Fig. S2. Data are means of four to six replicates; error bars are the SEM. Significance was determined using a *t*-test, **P<0.01.

Because a *PLUTO* knockout is able to synthesize HMP in the plastid, we created an *Arabidopsis py PLUTO* double knockout to test for reduced growth or a thiamin-deficient phenotype at various concentrations of exogenously supplemented HMP. The double mutant was not lethal at HMP concentrations needed to rescue the *py* mutant, suggesting that PLUTO is not the sole plastidial HMP transporter. As roots of various plants are known to have a limited capacity to synthesize thiamin [19–21], we checked for root growth phenotypes. In the absence of exogenous HMP, and at HMP concentrations below 10 nM, *py PLUTO* double mutant seedlings had significantly shorter roots than *py* single mutants (Figure 4 and Supplementary Fig. S2). Increasing the HMP concentration to 10 or 30 nM resulted in similar root growth of both mutants, indicating that PLUTO deficiency can be overcome by an excess of HMP.

## Discussion

All the thiamin biosynthetic enzymes and several salvage enzymes have been identified in *Arabidopsis*, but the only transporters identified are mitochondrial ThDP transporters [1,2]. As HMP-requiring mutants can be grown and propagated when HMP is exogenously supplied [4,7], the presence of HMP transporters on the plasma membrane and the plastid envelope can be strongly inferred. Here we show that PLUTO acts as a plastidial HMP transporter, albeit a partially redundant one.

Prokaryotic homologs of PLUTO, previously predicted to be HMP transporters and given the name CytX [15], occur in clusters with thiamin biosynthetic enzymes in several bacterial phyla. Furthermore, CytX is found in organisms that almost certainly cannot make HMP because they lack the biosynthetic enzyme ThiC. Additionally, the gene specifying CytX is often found in a cluster encoding ThiD (HMP kinase), ThiE (ThMP synthase), ThiM (HET kinase), and in some cases a putative HET transporter, which – if CytX transports HMP – would together provide all the machinery needed to make ThMP. It should, however, be noted that CytX is not the only prokaryotic HMP transporter [15].

PLUTO is known to mediate plastidial nucleobase transport in *Arabidopsis* [8,9], but it is also co-expressed with genes involved in thiamin metabolism. *Arabidopsis* PLUTO has been shown to transport uracil [8,9] and PLUTO homologs from other organisms have been shown to transport cytosine [22]; both uracil and cytosine are pyrimidines and are thus chemically similar to HMP. Additionally, *S. cerevisiae thi7* is structurally related to PLUTO [23] and transports thiamin [24].

Expressing PLUTO heterologously in *E. coli* or *S. cerevisiae* increased sensitivity to the HMP analog oHMP. oHMP is probably toxic because it can be phosphorylated by ThiD [18] and then converted to oxy-thiamin phosphates. However, it was shown not to be toxic in *E. coli*, likely due to lack of uptake [18]. The lack of an effective endogenous transporter for oHMP enables PLUTO-expressing and wild-type *E. coli* to be distinguished by exposing them to 1 mM oHMP (Figure 3A).

We could not demonstrate transport of [^3^H]HMP in these same *E. coli* strains due to a background uptake of HMP (Supplementary Fig. S1), presumably mediated by unidentified specific HMP transporters that do not transport oHMP (and hence do not interfere with oHMP toxicity tests). Many prokaryotic vitamin or vitamin precursor transporters have substrate affinities in the high picomolar to low nanomolar range [25], which mirrors the typical concentrations of these compounds in natural environments [26,27]. PLUTO has a *K*_m_ value in the micromolar range for uracil [8,9]. However, as cytosolic HMP concentrations are probably well above the trace levels found in the environment, PLUTO may not need a submicromolar affinity for HMP.

*Arabidopsis PLUTO* mutants show no thiamin deficiency symptoms [9], probably because the HMP moiety of thiamin is synthesized *de novo* in plastids (Figure 1), making HMP import dispensable [1]. If PLUTO is the sole plastidial HMP transporter, a *py PLUTO* double mutant should be lethal when HMP is supplied (but viable if thiamin is supplied). That the double mutant is not lethal and has only a moderate root growth defect (Figure 4) when HMP is supplied indicates that other plastidial transporters can act on HMP. This defect is unlikely to be due to a depletion of other PLUTO substrates such as uracil because the phenotype can be rescued with exogenous HMP. That the root growth defect of *py PLUTO* double mutant seedlings is apparent when no HMP is supplied (Figure 4) most likely reflects the partial dependence of these seedlings on salvaged HMP, which can come only from the action of extraplastidial enzymes (At3g16990 and At5g32470) that hydrolyze thiamin breakdown products [2,7]. Thus, the root phenotype establishes that PLUTO is a significant HMP transporter, at least in roots. In this connection, it is interesting that roots of various species have long been known to require exogenous thiamin or thiamin precursors for growth in culture and, *in planta*, to import these compounds from shoots [19–21]. While HMP and HET synthesis genes are expressed in *Arabidopsis* roots, their expression is weak [28] and spatially restricted [29]. This pattern is consistent with heavy reliance on HMP import into plastids to meet the demand for thiamin synthesis.

Finally, there is a clear parallel between our finding that the PLUTO plastidial nucleobase transporter doubles as an HMP transporter and the recent evidence that the *Arabidopsis* PUT3 polyamine transporter mediates phloem transport of both thiamin and polyamines [29]. Perhaps other unidentified transporters for B vitamins and their precursors [1] will likewise prove to be known transporters with moonlighting activities.

## Supporting information

**Supplementary Fig. S1.** IPTG-induced *E. coli* cells expressing PLUTO show no increase in HMP uptake when supplied with 12.5 nM [^3^H]HMP relative to an empty vector control (EV). *E. coli* expressing PLUTO (black circles) or the empty vector control (white circles) were grown as described [7] and resuspended to an OD^600^ of 10 in M9-glucose medium. Assays were initiated by combining 500μL of cell suspension and 500μL of M9-glucose containing 25 nM [^3^H]HMP. Aliquots (50μL) were taken, the cells separated by filtration, washed, and [^3^H]HMP uptake measured by liquid scintillation counting. Data are means and SEM for three independent replicates.

**Supplementary Fig. S2.** Representative *py* and *py PLUTO* Arabidopsis mutants grown with or without exogenous HMP. Plants were grown for 6 days on ½ MS medium with sucrose.

**Supplementary Table S1.** GeneMANIA query and co-expressed genes

## Abbreviations

HET: hydroxyethylthiazole
HMP: hydroxymethylpyrimidine
oHMP: oxy-HMP
PLUTO: plastidic nucleobase transporter
*py*: pyrimidine-requiring
ThDP: thiamin diphosphate
ThMP: thiamin monophosphate
