# Supplementary material for: The PLUTO plastidial nucleobase transporter also transports the thiamin precursor hydroxymethylpyrimidine
Source: Biosci Rep. 2018 Mar 29;38(2):BSR20180048. doi: 10.1042/BSR20180048 (PMC5874261; doi:10.1042/BSR20180048)
Supplement: Supplementary file 1 [file bsr20180048_Supp1.pdf]

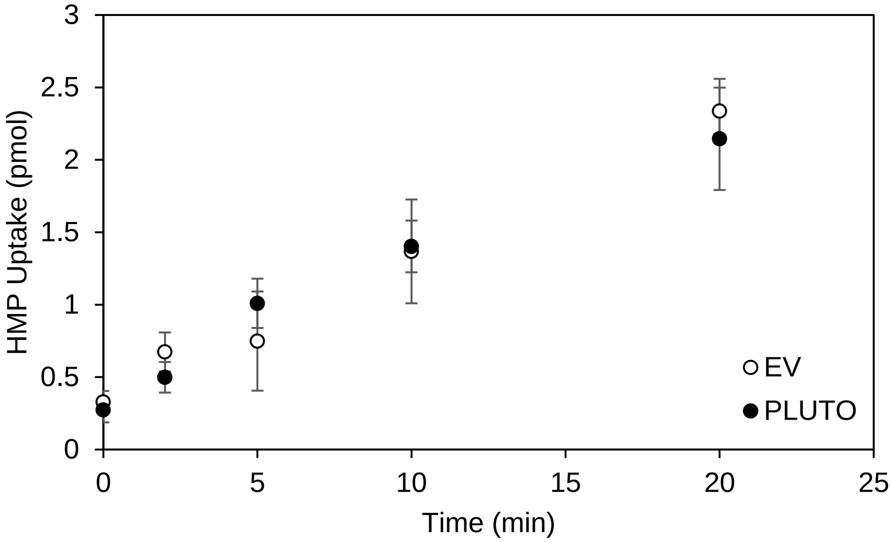

Supplementary Fig. S1. IPTG-induced *E. coli cells* expressing PLUTO show no increase in HMP uptake when supplied with 12.5 nM [^{3}H]HMP relative to an empty vector control (EV). *E. coli* expressing PLUTO (black circles) or the empty vector control (white circles) were grown as described [7] and resuspended to an OD_{600} of 10 in M9-glucose medium. Assays were initiated by combining 500 µL of cell suspension and 500 µL of M9-glucose containing 25 nM [^{3}H]HMP. Aliquots (50 µL) were taken, the cells separated by filtration, washed, and [^{3}H]HMP uptake measured by liquid scintillation counting. Data are means and SEM for three independent replicates.

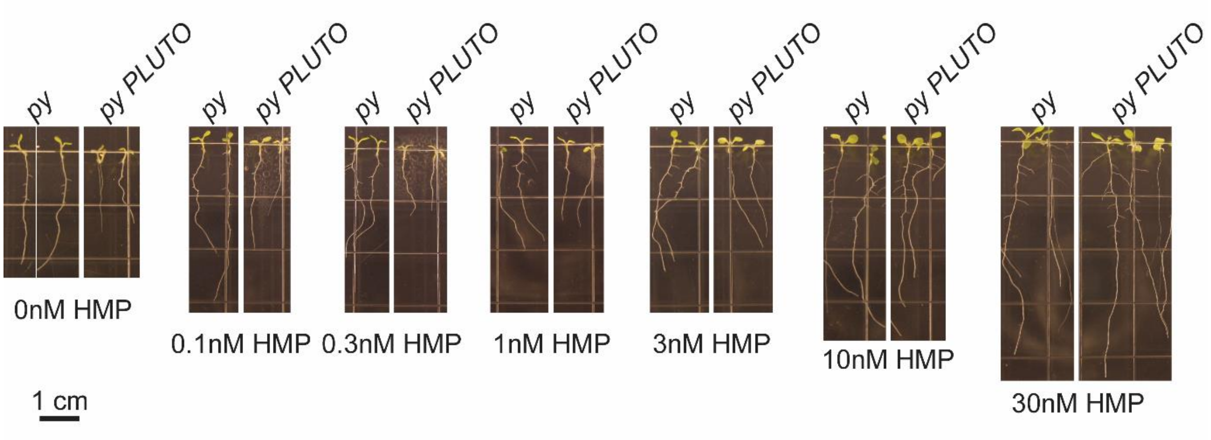

Supplementary Fig. S2. Representative *py* and *py PLUTO Arabidopsis* mutants grown with or without exogenous HMP. Plants were grown for 6 days on ½ MS medium with sucrose.

Supplementary Table S1. GeneMANIA query and co-expressed genes

	Gene name	Locus tag	Description
Query genes	NCS1	*At5g03555*	Nucleobase cation symporter 1
	THIC	*At2g29630*	HMP-P synthase
	TH1	*At1g22940*	HMP-P/HMP kinase; Thiamin-phosphate pyrophosphorylase
	THI1	*At5g54770*	Thiazole synthase
	At1g02880	*At1g02880*	Thiamin pyrophosphokinase
	At2g44750	*At2g44750*	Thiamin pyrophosphokinase
	THIM	*At3g24030*	HET kinase
	TNEA_C	*At5g32470*	Thiamin monophosphate hydrolase
	TENA_E	*At3g16990*	Thiaminase
	NUDT20	*At5g19460*	Nudix hydrolase 20, associated with thiamin pyrophosphokinase
	NUDT24	*At5g19470*	Nudix hydrolase 24, associated with thiamin pyrophosphokinase
	COG0212	*At1g76730*	5-Formyltetrahydrofolate cyclo-ligase-like protein
	At5g48970	*At5g48970*	Mitochondrial thiamine diphosphate transporter TPC1
	At3g21390	*At3g21390*	Mitochondrial thiamine diphosphate transporter TPC1
Co-expressed genes	PHS2	*At3g46970*	Alpha-glucan phosphorylase 2, cytosolic
	TRY	*At5g53200*	Transcription factor TRY
	At1g64430	*At1g64430*	Pentatricopeptide repeat (PPR) superfamily protein
	At4g24700	*At4g24700*	unknown protein
	At5g17570	*At5g17570*	TatD related DNase
	GCN2	*At3g59410*	Probable serine/threonine-protein kinase GCN2
	At4g00490	*At4g00490*	Beta-amylase 2
	SPA4	*At1g53090*	Protein SPA1-RELATED 4
	AHL	*At5g54390*	PAP-specific phosphatase HAL2-like
	At4g09620	*At4g09620*	Mitochondrial transcription termination factor family protein
	RPP13	*At3g46530*	Disease resistance protein RPP13
	At1g78995	*At1g78995*	unknown protein
	At5g02830	*At5g02830*	Pentatricopeptide repeat-containing protein
	T2E22.34	*At3g12320*	unknown protein
	LCAT1	*At1g27480*	Lecithin-cholesterol acyltransferase-like 1
	SBE2.1	*At2g36390*	1,4-Alpha-glucan-branching enzyme 2-1
	At5g40060	*At5g40060*	Disease resistance protein (NBS-LRR class) family
	MT2A	*At3g09390*	Metallothionein-like protein 2A
	EPF1	*At2g20875*	Protein EPIDERMAL PATTERNING FACTOR 1
	At5g62130	*At5g62130*	Per1-like family protein
